# Supplementary figures and images for: Correlative fluorescence microscopy, transmission electron microscopy and secondary ion mass spectrometry (CLEM-SIMS) for cellular imaging
Source: PLoS One. 2021 May 10;16(5):e0240768. doi: 10.1371/journal.pone.0240768 (PMC8109779; doi:10.1371/journal.pone.0240768)

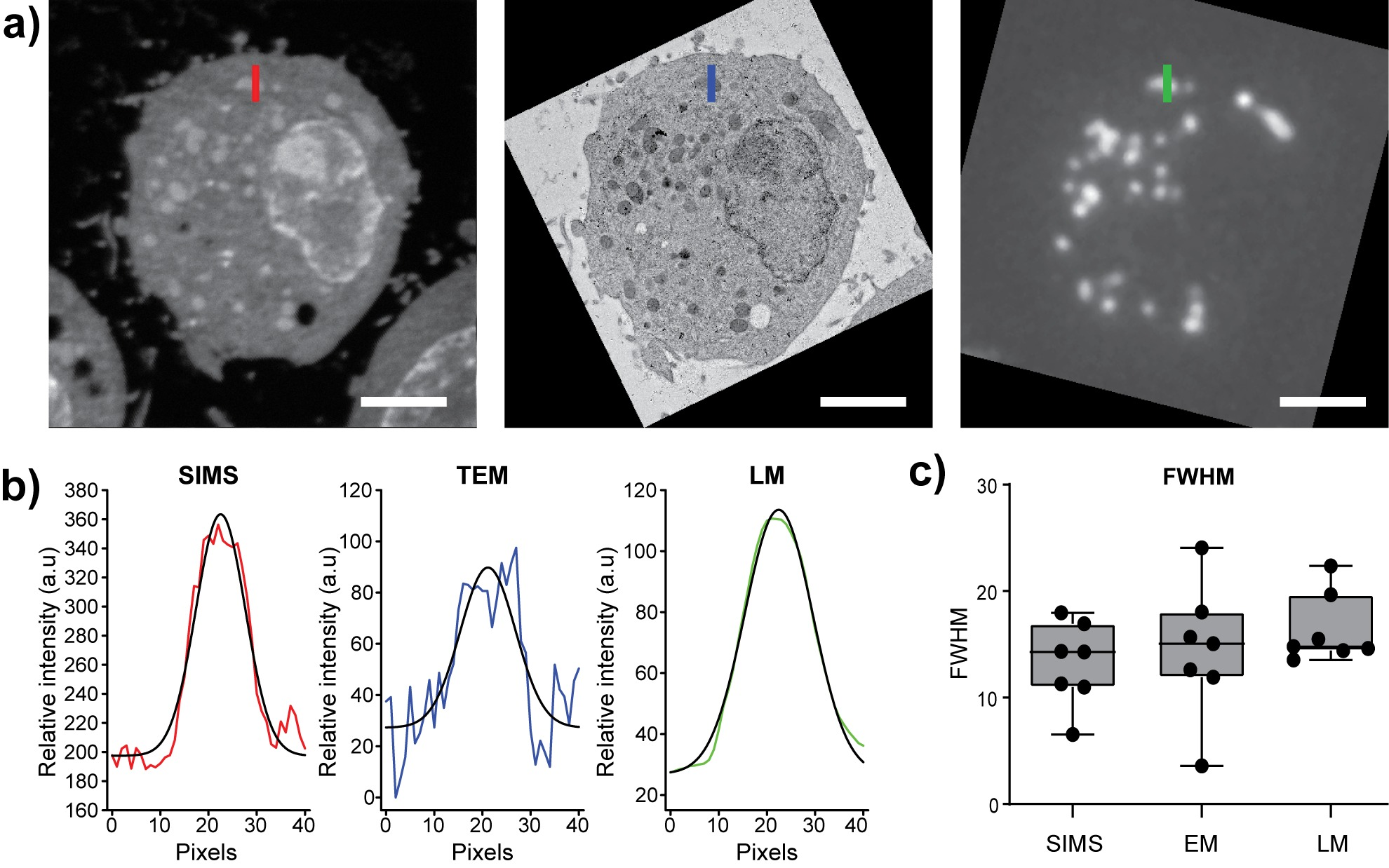

Supplement: S1 Fig — a) The same cell was imaged, from left to right, in nanoSIMS, TEM, and LM. Scale bar: 5 μm. b) Line profiles were drawn across the mitochondrion indicated by the color lines in panel a, and were fitted to Gaussian curves, to determine the full width at half maximum (FWHM) of the objects. c) An analysis of FWHM in mitochondria selected in multiple cells. For simplicity, the FWHM was left in number of pixels. No statistically significant differences could be detected using a Kruskal-Wallis test (p = 0.3384). The middle line in the boxes indicates the median, while the boxes show the 25th percentiles; the error bars show the full range of values percentiles, with symbols indicating all measurement values. (TIF) [file pone.0240768.s001.tif]

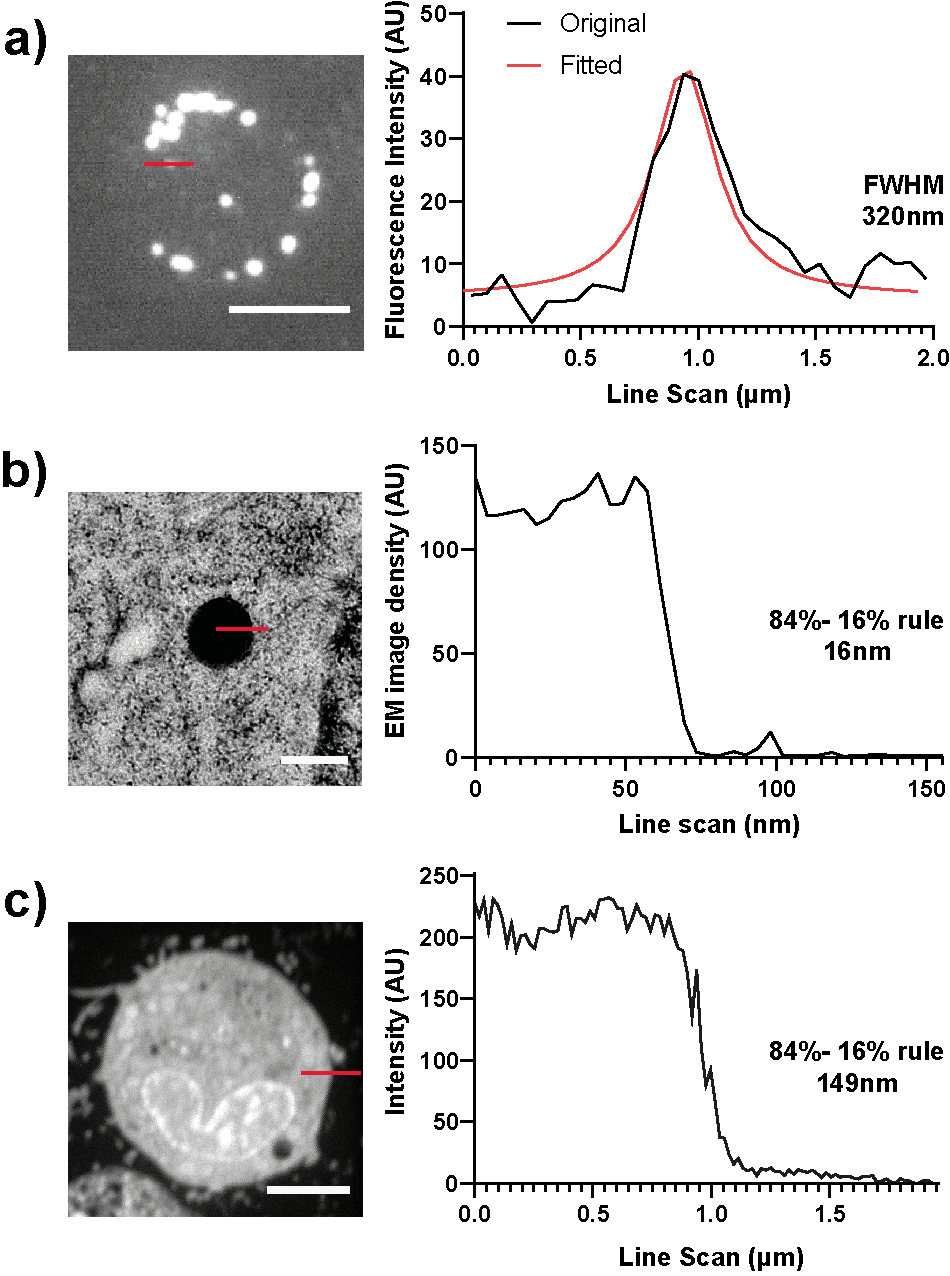

Supplement: S2 Fig — a) An analysis of the fluorescence imaging resolution. Line profiles were drawn across small spots in the MitoTracker images (same image as in Fig 4). The full width at half maximum (FWHM) was then determined from Gaussian curves fitted on the spots. The average resolution is 321 ± 36 nm (mean ± SD, from 10 different measurements). Scale bar: 5 μm. b) A similar analysis for TEM images. To determine the lateral resolution, the distance in which the intensity of the signal drops from 84% to 16% of the maximum is calculated. The average resolution is 22 ± 6 nm (mean ± SD, from 10 different measurements). Scale bar: 500 nm. c) A similar analysis for SIMS images, relying on 12C14N images, and using the 84%-16% resolution measurement (same image as in Fig 4). The average resolution is 164 ± 27 nm (mean ± SD, from 7 different measurements). Scale bar: 5 μm. (TIF) [file pone.0240768.s002.tif]

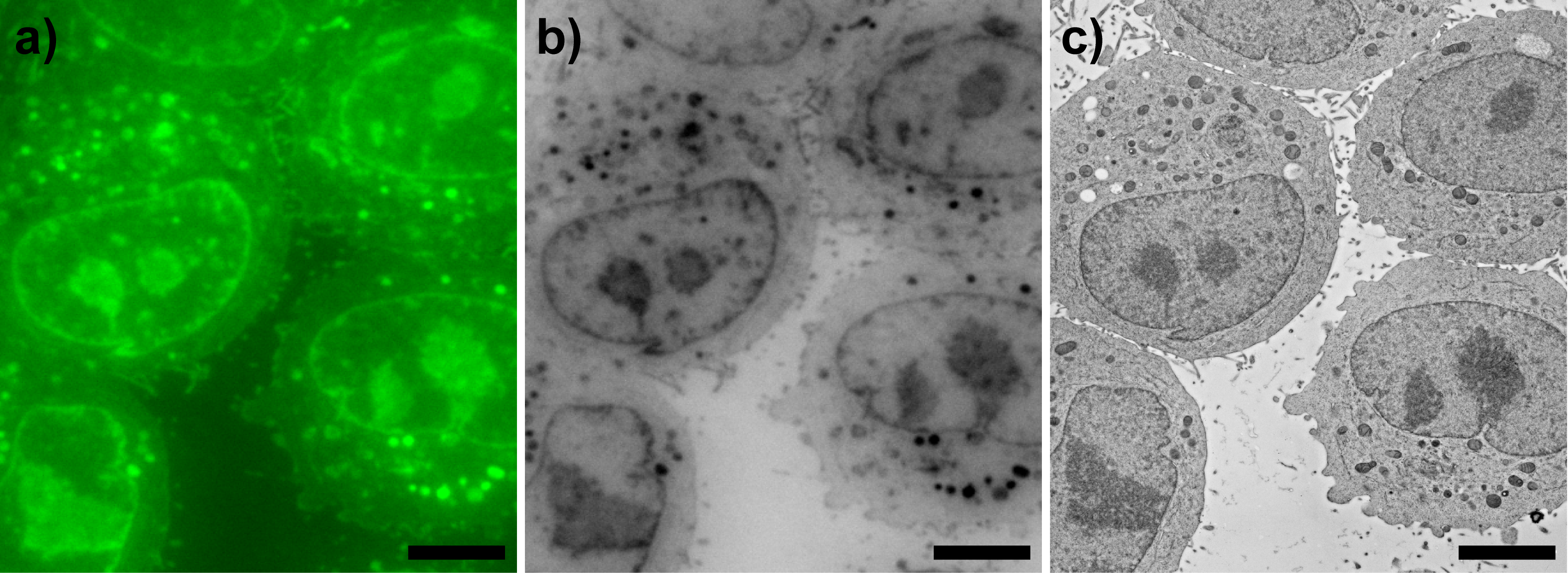

Supplement: S3 Fig — (TIF) [file pone.0240768.s003.tif]
